# Correlates of institutional deliveries among teenage and non-teenage mothers in Nepal

**DOI:** 10.1371/journal.pone.0185667

**Published:** 2017-10-11

**Authors:** Pawan Acharya, Tara Ballav Adhikari, Dipika Neupane, Kiran Thapa, Parash Mani Bhandari

**Affiliations:** 1 Young Earth, Kathmandu, Nepal; 2 Unit for Health Promotion Research, University of Southern Denmark, Esbjerg, Denmark; 3 Maharajgunj Medical Campus, Institute of Medicine, Kathmandu, Nepal; Helsingin Yliopisto, FINLAND

## Abstract

**Introduction:**

Globally, maternal age is identified as an important predictor of institutional service utilization during delivery. This study aims to assess the correlates of institutional delivery among teenage and non-teenage mothers in Nepal by using the data from Nepal Demographic and Health Survey 2011.

**Methods:**

The study population consisted of 5391 women of reproductive age (15–49 years) who had given birth to a child within five years before the survey. Out of them, 381 (7.07%) were teenage mothers. The association between the background characteristics and institutional delivery was assessed separately for the teenage and non-teenage mothers using chi-square test and multiple logistic regression analysis.

**Results:**

After adjusting for background characteristics, teenage mothers were found more likely to deliver at a health facility [AOR: 2.25; 95% CI: 1.10 4.59] in comparison to the non-teenage mothers. Place of residence, occupation, socioeconomic status, and frequency of ANC visits were associated with institutional delivery in both the teenage and non-teenage mothers. However, educational status, parity, birth preparedness and women autonomy had statistically significant association with institutional delivery among the non-teenage mothers only. None of the background characteristics were significantly associated with institutional delivery in teenage mothers only.

**Conclusions:**

This study identified a significant difference in institutional delivery service utilization among the teenage and non-teenage mothers. While the association of most of the background characteristics with institutional delivery was uniform for both teenage and non-teenage mothers, the association with educational status, parity, birth preparedness and women autonomy was significant only for non-teenage mothers. Considering this difference in the interaction of women’s background characteristics with institutional delivery between teenage and non-teenage mothers might help in identifying the pain points and devise targeted interventions to encourage institutional delivery in teenage mothers or non-teenage mothers or both.

## Introduction

Despite the notable progress in reducing maternal deaths around the world by 43% over 25 years since 1990, approximately 830 women die every day due to perinatal complications [1]. Nearly 99% of these deaths occur in developing countries like Nepal.

Acknowledging the need for programs to reduce preventable maternal deaths, the Government of Nepal (GoN) has implemented programs like user fee exemption, travel incentives for delivery at health facilities, and provision of 24-hour emergency obstetric services to enhance institutional deliveries by skilled birth attendants. To encourage delivery at health facilities and make pregnancy and childbirth safer, health posts are being gradually upgraded to include birthing centers [2]. As a result, some progress has been achieved. Nepal has been successful in increasing institutional delivery from 8% to 35% in between 1996 and 2011 [3,4], and in reducing maternal mortality ratio from 539 to 190 per 100,000 live births in between 1995 and 2015 [5].

Still, several challenges remain in further reducing the maternal deaths. The remoteness of health facilities is one of the challenges. Health institutions are inaccessible in many rural sites and the available health services are underutilized [6]. A recent study linked longer distance of hospital from home with unaccounted costs that possibly prevent utilization of health services in rural Nepal, especially among poor households [7].

About 17 percent of adolescent women aged 15–19 years are already mothers or pregnant with the first child. Majority of newly married women are teenagers as reflected by the median age at marriage among women aged 25–49 years [4]. In these young mothers, poor pregnancy outcomes and negative social consequences are common. Teenage mothers who find themselves biologically fit to deliver a baby may be struck by a myriad of problems that range from marital instability, sexual harassment to depressive symptoms [8,9]. Compared with older women, teenage mothers are at higher risk of maternal death [10] but are less likely to use maternal health services [11]. In response to these specific needs of teenage mothers, GoN has developed the National Reproductive Health Strategy 1998 and has integrated adolescent sexual and reproductive health issues in national long-term program and plans in a row. Despite these policy and programmatic level plans and commitments, reduction in teenage pregnancy rate is very slow and Nepal still has one of the highest teenage pregnancy rates in South Asia [12,13]. For these reasons, teenage pregnancy remains a huge challenge in further reduction of maternal mortality in Nepal.

In terms of research, the effect of mother’s age on the utilization of maternal health services has been studied frequently; however, the determinants of maternal health service utilization in teenage and non-teenage mothers have not been compared often. Aggregated data fail to provide a lucid picture of the differential needs and situation of teenage mothers. Considering the diverse culture, socioeconomic status, and ethnicity of Nepalese society, it becomes more necessary to explore the potential determinants of institutional delivery among teenage and non-teenage mothers and their difference. So, in this study, we aimed to identify the factors associated with institutional delivery among teenage and non-teenage mothers in Nepal.

## Material and methods

**Fig 1:**
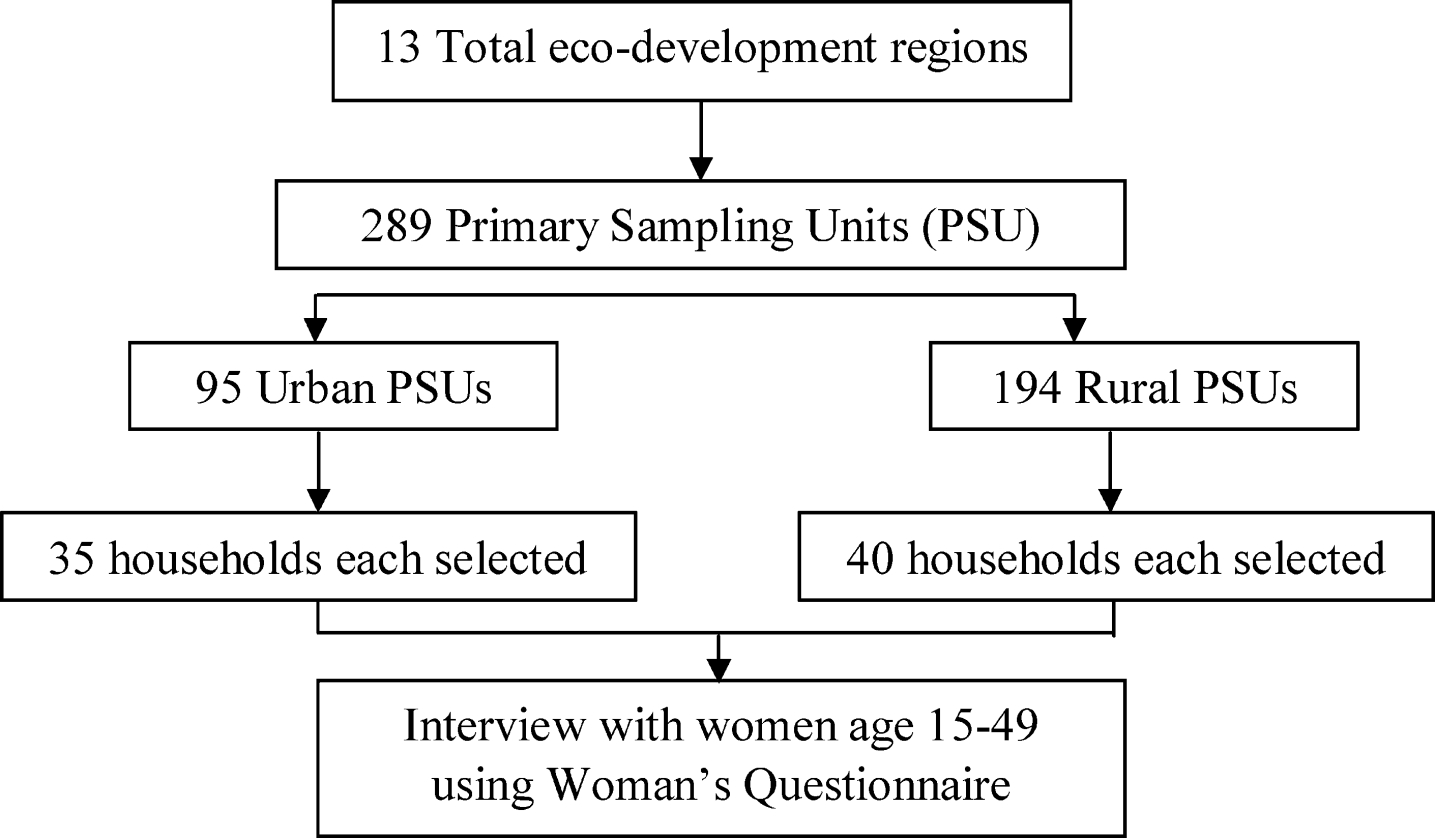
Flowchart depicting selection of mothers interviewed.

This study used data from Nepal Demographic and Health Survey (NDHS) 2011. NDHS was conducted based on a two-stage cluster sampling technique. The country was at the first divided into 13 sample domains and 25 sample strata. At the second stage, 289 primary sampling units (PSU) were created. Then, households were selected randomly from the PSU according to probability-proportionate-to-size technique. (Fig 1)

Details of the survey and sampling procedure have been published in the original report [4]. Total 5391 women of reproductive age (15–49 years), who had become pregnant within five years before the survey, were included in this analysis.

### Definitions of variables

Two specific geographical variables were included—Place of residence (‘Urban’, ‘Rural’) and Ecological zone (‘Mountain’, ‘Hill’, ‘*Terai*’). Here, *Terai* refers to the plain ecological belt of Nepal that lies in the South. Respondent’s and her husband’s education status was categorized into ‘No education’, ‘Primary education’ and ‘Secondary education or higher’. Participant’s occupation was categorized as ‘Not working’, ‘Agriculture’ and ‘Paid jobs’. Ethnicity was first classified into seven groups according to the classification criteria used by NDHS [14] and then regrouped into four categories: ‘Advantaged ethnicities’ comprising *Brahmin and Chhetri*, ‘Relatively disadvantaged ethnicities’ including *Newar*, *Janajati*, *Madheshi and Muslims*, ‘Disadvantaged ethnicities’ consisting *Dalits*, and ‘Other ethnic minorities’ consisting of unspecified and unidentified ethnic groups. This categorization is frequently used in other reports on the further analysis of NDHS data [15–17]. It has to be noted that ethnic group mainly represents the traditional social hierarchy mainly in the caste system and might not necessarily represent the social class. Similarly, the socioeconomic group was derived from wealth quintile; wealth quintile was calculated from household assets using principal component analysis [18] and was divided into five equal categories in rank (poorest, poorer, middle, richer and richest) each comprising 20% of the population. The quintiles were further merged into three categories to form economic status variable- the lowest 40% (poorest and poor) as ‘Poor’; middle 40% (middle and rich) as ‘Middle’; and the upper 20% (richest) as ‘Rich’, as done by previous researchers [16,19,20].

Some women-specific behavioral and fertility-related factors included in this study were–number of Antenatal Care (ANC) visits by the woman (‘None’, ‘1–3 visits’, 4 or more visits’), parity (‘Primi’, ‘Multi’), pregnancy intentness and level of birth preparedness. As recommended by the GoN, birth preparedness consists of the family saving money for emergencies, arranging transportation facilities best for the locality, identifying suitable blood donors, identifying and contacting health workers who can provide maternity services, and having a clean delivery kit handy [21].

Similarly, women autonomy was derived from the information obtained during the interview, mainly regarding the woman’s participation in the household decision making on spending the money earned, purchasing household goods/property, visiting a friend/relative and ability to make decisions regarding her health care. Autonomy was categorized into- ‘No autonomy’: if the woman did not participate at all in the decisions made in the family, ‘Partial autonomy’: if the woman had a say in the decisions, and ‘Full autonomy’: if the woman could decide solely on all the above-mentioned issues.

### Statistical analysis

Statistical analysis was performed on STATA 14.0 using survey analysis technique. The association between background characteristics and the utilization of institutional delivery services by the teenage and non-teenage woman was assessed separately by calculating chi-square test for each group. Adjusted odds ratio and its 95% confidence interval (CI) were calculated, while statistically adjusting for background characteristics, separately for both age groups by multiple logistic regression analysis. A stepwise backward elimination method was deployed to select the variables to be included in the multiple logistic regressions. A cut–off of p = 0.05 was chosen and the variables significantly associated (p<0.05) with institutional delivery were selected to be included in the final multiple regression analysis. To address the false discovery rate, we deployed Benjamini-Hochberg procedure [22] to calculate the adjusted p-values.

### Ethical clearance

This study was based on the secondary data from NDHS 2011. The NDHS obtained ethical approval from Nepal Health Research Council, Kathmandu, and Macro Institutional Review Board, Maryland, USA [4]. Informed consent was obtained from the respondents (women of reproductive age and their husband) to participate in the survey before the interview was conducted. A special statement was included in the beginning of the household and the individual questionnaire. This statement explained the purpose of the study. Participation in the survey was completely voluntary and the respondent was informed that s/he had the right to refuse to answer any questions or stop the interview at any point. The informed consent statement was read exactly as it was written before the respondent was asked to participate in the interview. Then the interviewer signed his or her name attesting to the fact that s/he read the consent statement to the respondent. However, no written consent was obtained from the respondents. Details on the ethical measures employed during the survey are explained elsewhere [4,23]. Measure DHS program permitted access to the secondary data for this study.

## Results

### Participant characteristics

Distribution of institutional delivery among the teenage and non-teenage mothers according to their background characteristics is shown in Table 1. (Table 1)

**Table 1:** Distribution of teenage and non-teenage mothers delivered at the health facility according to their background characteristics-NDHS 2011 (N = 5391).

	Sample						Institutional delivery	
Characteristics	Total		Non-teenage mothers (n = 5010)		Teenage mothers (n = 381)		Non-teenage mothers (n = 5010)	Teenage mothers (n = 381)
	n	%	n	%	n	%	%	%
**Place of residence**							**P<0.001**	**P<0.001**
Urban	503	9.34	479	9.56	24	6.42	70.86	80.6
Rural	4888	90.66	4531	90.44	357	93.58	30.32	48.21
**Ecological zone**							**P<0.001**	**P<0.001**
Mountain	428	7.93	400	7.99	27	7.18	17.48	38.72
Hill	2130	39.51	2001	39.95	129	33.76	31.01	35.57
*Terai*	2833	52.55	2608	52.06	225	59.06	39.21	60.11
**Education**							**P<0.001**	**P = 0.032**
No education	2549	47.29	2456	49.03	93	24.42	18.23	48.48
Primary education	1079	20.02	964	19.25	115	30.08	30.63	38.79
Secondary education or higher	1763	32.69	1589	31.72	173	45.5	61.05	58.85
**Occupation**							**P<0.001**	**P<0.001**
Not working	1553	28.80	1396	27.87	156	41.04	50.19	63.51
Agriculture	3692	68.49	3471	69.29	221	58.01	26.28	40.12
Paid jobs	146	2.71	142	2.84	4	0.95	70.25	100
**Husband’s education**							**P<0.001**	**P = 0.166**
No education	1244	23.07	1176	23.48	67	17.66	15.34	41.83
Primary education	1311	24.31	1214	24.24	96	25.29	23.31	49.18
Secondary education or higher	2801	51.95	2589	51.67	212	55.61	48.12	54.78
Don't know	36	0.67	31	0.61	5	1.44	12.78	0
**Ethnicity**^**#**^							**P<0.001**	**P = 0.579**
Advantaged ethnicities	1618	30.02	1552	30.97	67	17.49	43.77	51.61
Relatively disadvantaged ethnicities	2243	41.61	2080	41.52	163	42.73	30.88	45.52
Disadvantaged ethnicities	959	17.78	860	17.16	99	25.93	23.38	52.95
Other ethnic minorities	571	10.60	519	10.35	53	13.84	36.77	58.33
**Socioeconomic status**							**P<0.001**	**P<0.001**
Poor	2572	47.71	2389	47.69	183	47.93	15.44	36.02
Middle	2071	38.41	1900	37.92	171	44.91	41.44	58.42
Rich	748	13.88	721	14.39	27	7.16	77.27	94.79
**Parity**							**P<0.001**	**P = 0.233**
Primi	1832	33.99	1499	29.92	333	87.52	54.59	51.73
Multi	3559	66.01	3511	70.08	48	12.48	25.49	40.17
**Pregnancy intentness**							**P<0.001**	**P = 0.044**
Intended	4065	75.41	3797	75.78	269	70.54	36.16	46.09
Unintended	1326	24.59	1213	24.22	112	29.46	28.06	60.33
**ANC visit (n = 4149)**							**P<0.001**	**P = 0.010**
No visit	629	11.66	602	15.77	27	8.13	7.65	21.6
1–3 visits	1442	26.74	1316	34.51	125	37.58	21.12	50.34
4 or more visits	2078	38.54	1898	49.72	181	54.29	57.58	62.02
**Birth preparedness**							**P<0.001**	**P = 0.012**
None	2609	48.40	2415	63.3	194	58.29	26.82	47.16
One	1382	25.64	1251	32.8	131	39.38	52.09	62.28
Two or more	157	2.90	148	3.9	8	2.32	78.48	100
**Women autonomy**^**$**^							**P<0.001**	**P = 0.354**
No autonomy	1424	26.76	2076	42.00	97	25.58	27.81	48.66
Partial autonomy	1984	37.27	1936	39.17	141	37.12	34.63	55.79
Full autonomy	1915	35.98	931	18.84	142	37.29	37.86	42.09

^#^Ethnicity is classified into three categories: ‘Advantaged’ category includes *Brahmin and Chhetri* ethnicities. Similarly, ‘Relatively disadvantaged’ group includes *Janajatis*, *Newar and Muslims* and the third category–‘Disadvantaged’ consists *Dalits*. Other unidentified ethnic groups were kept separately as ‘Other ethnic minorities’.

^$^Some missing values (n = 5322)

Among the 5391 women delivered within five years before the survey, 7.07% were below 20 years of age. More than 90% were from rural areas and the majority of them (52.55%) were from *Terai* ecological region. About 47% had no education and more than two-third (68.49%) worked in agriculture. More than half (51.95%) of the women's husband had secondary or higher education. Ethnically, every two in five (41.61%) belonged to the relatively disadvantaged ethnic group. About 48% of the respondents belonged to the poor socioeconomic group. More than three-quarters of the mothers (75.41%) mentioned that their last-born child was intended. (Table 1)

In the unadjusted model, the odds of institutional delivery was significantly higher among the teenage mothers [OR: 1.94; 95% CI: 1.49 2.54] in comparison to their non-teenage counterparts. After adjusting the model for background characteristics, the odds ratio further increased [AOR: 2.25; 95% CI: 1.10 4.59]. (Table 2)

**Table 2:** Association between age groups and institutional delivery-NDHS 2011 (N = 5391).

Age group	n	%	OR	[95% CI]		AOR^1^	[95% CI]	
>20 years (n = 5010)	1713	34.19	1.00			1.00		
<20 years (n = 381)	189	49.71	1.94	[1.49	2.54]***	2.25	[1.10	4.59]*

*p<0.05

***p<0.001

OR: odds ratio; AOR: adjusted odds ratio; CI: confidence interval

^1^ Adjusted for place of residence, woman’s education, woman’s occupation, socioeconomic status, number of ANC visits, birth preparedness and the interaction term for maternal age group and education status

We assessed the interaction among the socio-demographic variables; however, there was no statistically significant interaction, except for maternal age group and education. The AOR in Table 2 is adjusted for the interaction between maternal education status and age group. The multiple logistic model, in overall, was highly significant (p<0.0001). Similarly, an exploratory check of our aggregate model (keeping both teenage and non-teenage mothers together) was done, using primary sampling units as random variables. In the empty model (without any independent variables), the community level variance was 2.19 (SE = 0.29) whereas, in the final aggregate model, the community level variance was 0.53 (SE = 0.16). The proportion change in variance was 75.79 percent, meaning that about 76% of the variation in the odds of institutional delivery between communities was explained by the background characteristics included in the multiple logistic regression model. However, this study aims to assess the effect of individual background characteristics on institutional delivery utilization and therefore, only the fixed effects will be discussed further.

### Factors associated with institutional deliveries

Being a rural resident was found to be negatively associated with institutional delivery among both the teenage [AOR: 0.46; 95% CI: 0.24 0.89] and non-teenage [AOR: 0.42; 95% CI: 0.31 0.57] mothers. (Table 3)

**Table 3:** Factors associated with the institutional delivery among teenage and non-teenage mothers in Nepal- NDHS 2011 (N = 5391).

	Teenage mothers			Non-teenage mothers		
Characteristics	AOR	[95%CI]		AOR	[95%CI]	
**Place of residence**						
Urban	1.00			1.00		
Rural	0.46	[0.24	0.89]*	0.42	[0.31	0.57]***
**Mother's education**						
No education				1.00		
Primary education				1.22	[0.93	1.61]
Secondary education or higher				1.80	[1.39	2.32]***
**Mother’s occupation**						
Not working	1.00			1.00		
Agriculture	0.42	[0.21	0.86]*	0.57	[0.45	0.72]***
Paid jobs	1.00			0.67	[0.37	1.23]
**Socioeconomic status**						
Poor	1.00			1.00		
Middle	1.73	[1.00	3.02]*	1.98	[1.54	2.54]***
Rich	13.70	[1.89	59.09]*	4.71	[3.35	6.64]***
**ANC visit**						
No visit	1.00			1.00		
1–3 visits	3.53	[1.26	9.89]*	1.94	[1.17	3.20]*
4 or more visits	5.04	[1.91	13.29]**	5.25	[3.13	8.78]***
**Parity**						
Primi				1.00		
Multi				0.40	[0.32	0.50]***
**Birth preparedness**						
None				1.00		
One				1.70	[1.35	2.14]***
Two or more				3.72	[2.16	6.41]***
**Women autonomy**						
No autonomy				1.00		
Some autonomy				1.15	[0.90	1.47]
Full autonomy				1.30	[1.02	1.65]*
**Intercept**	0.72	[0.21	2.46]	0.34	[0.19	0.61]***

AOR: adjusted odds ratio; CI: confidence interval

*<0.05

**<0.01

***<0.001

Among non-teenage mothers, the odds of institutional delivery was significantly higher [AOR: 1.80; 95% CI: 1.39 1.23] if the mothers had a secondary or higher education.

Mothers working in the agricultural sector compared to non-working mothers had lower odds of institutional delivery in both teenage [AOR: 0.42; 95% CI: 0.21 0.86] and non-teenage [AOR: 0.57; 95% CI: 0.45 0.72] groups. Higher odds of institutional delivery were found among a higher socioeconomic group within both age groups. Among teenage mothers, the odds of institutional delivery was higher at the marginally significant level (p = 0.05) among the middle [AOR: 1.73; 95% CI: 1.00 3.02] and rich [AOR: 13.70; 95% CI: 1.89 59.09] economic groups. A similar pattern was found among non-teenage mothers across the economic groups where the odds for middle and higher socioeconomic status were about two-fold [AOR: 1.98; 95% CI: 1.54 2.54] and five-fold [AOR: 4.71; 95% CI: 3.35 6.64] higher respectively.

The frequency of ANC visits was found positively correlated with the institutional delivery in both age groups. Among the teenage mothers, the odds of institutional delivery was higher among those who visited ANC 1–3 times [AOR: 3.53; 95% CI: 1.26 9.89] or 4 times [AOR: 5.043; 95% CI: 1.91 13.29] in comparison to no ANC visits. Among the non-teenage mothers too, it was almost two-fold [AOR: 1.94; 95% CI: 1.17 3.20] for those who visited ANC 1–3 times and five-fold [AOR: 5.25; 95% CI: 3.13 8.78] for 4 or more ANC visits.

Multiparity was found to be associated with lower odds of institutional delivery among non-teenage mothers [AOR: 0.40; 95% CI: 0.32 0.50], whereas, the association was non-significant among teenage mothers.

Birth preparedness was found to have a positive effect on institutional delivery among the non-teenage mothers. Presence of at least one birth preparedness component was associated with higher odds [AOR: 1.70; 95% CI: 1.35 2.70] of institutional delivery among non-teenage mothers while birth preparedness had no significant effect on institutional delivery among teenage mothers. Similarly, women autonomy had rather small but positive effect on institutional delivery, however, the association was only significant among the non-teenage mothers [AOR: 1.30; 95% CI: 1.02 1.65].

## Discussion

This study was carried out to understand the influence of age on the utilization of institutional facilities and to assess factors associated with institutional delivery among teenage and non-teenage mothers separately. This study found that teenage mothers were more likely to deliver in health institution than non-teenage mothers. Other factors consistently associated with institutional delivery among both age groups were the place of residence, occupation, socioeconomic status, and frequency of ANC visits. However, educational status, parity, birth preparedness and women autonomy were found significantly associated with institutional delivery among non-teenage mothers only.

This study found higher odds of institutional delivery among teenage mothers than non-teenage mothers. No association of maternal age with the utilization of the institutional delivery service was observed in an earlier study carried out in Chitwan district of Nepal by Shah et al [24]. Evidence on the relation of maternal age with the site of delivery is inconsistent: while an earlier study from Ethiopia suggested no association [25], Nepal Demographic and Health Survey 2001 and 2006 consistently reported a greater percentage of teenage population delivering in a health facility [26,27]. Pregnancy in teenage women is often riskier than that in non-teenage women because of the underdeveloped pelvic organs [28]. Often, women with high-risk pregnancy are the ones who perceive themselves at risk [29]. This risk perception might have contributed to higher rates of institutional deliveries among teenage women.

Mothers residing in rural areas had lower odds of institutional delivery for both age groups. This might be because of the long distance to reach the health facility, difficult transportation and substandard quality of maternity care services at the poorly staffed rural health centers of Nepal [24,30]. According to a study from Western Nepal, distant health service is linked with added unaccounted costs [7], which can discourage poor pregnant women from seeking delivery in health institutions.While the health institutions in urban locations are relatively well-equipped in terms of health workers and transportation facility, most of the rural settlements of Nepal do not share these facilities [31,32]. To increase access to health service, outreach clinics providing maternal and child health services at a location flexible to most people have been introduced by GoN. These mobile clinics might be an alternative to establishing a permanent health service delivery site in these rural sites. However, no deliberate study has been carried out till date to compare utilization and cost-effectiveness of these outreach clinics. Beside these regular outreach clinics, health camps targeting these hard-to-reach areas are also conducted [33]; but these camps are infrequent and sporadic in nature.

Furthermore, the result of our study showed a positive influence of socioeconomic status on institutional delivery among both teenage and non-teenage mothers. Studies from Nigeria and Pakistan have also reported a positive association between socioeconomic status and institutional delivery [34,35]. Wagle et al. in their study among women from Central Nepal observed that likelihood of home delivery is high for women with low amenity score [36]. Hospital care, including medicines in the Essential Medicine List (list of medicines pre-specified by the GoN), is free in the district public hospitals and peripheral public health facilities. However, people have to make out-of-pocket payments for health services- including maternity care- in the tertiary and other private health facilities. GoN is trying to promote institutional deliveries in Mountainous, Hilly and *Terai* region by providing NPR 1500, 1000 and 500 respectively for women undergoing institutional delivery. However, this remuneration appears to be inadequate given the formal payment alone for stay at a tertiary maternity hospital averaged NPR 1965 according to a study in Kathmandu [37]. In addition to the ‘formal payment’, this allowance might also be inadequate to compensate for the hidden costs of seeking institutional delivery in rural areas [7].

The findings of our study suggested no significant influence of husband’s educational status on institutional delivery among both teenage and non-teenage mothers, which is contradictory to other research findings [25,35]. But, this might be a true reflection with regard to Nepalese population where the father being well-educated or illiterate might not do much on decision making when a woman gives birth to her child. We can only speculate as no such findings have been established by this study. Pregnant women’s educational status, however, showed significantly positive influence among non-teenage mothers. Similar findings are demonstrated in other studies as well [34,38]. This may be due to the fact that education makes women more aware of the risks of delivery at home and benefits of delivery at a health institution. It means that perceived need for institutional care is created among non-teenage mothers who otherwise were satisfied with delivery at home. Intriguingly, teenage mother’s utilization of delivery facility which was already higher than that of non-teenage mothers was not influenced by their educational status.

In this study, we found an interesting association between women’s occupation and institutional delivery. The pregnant women involved in agricultural occupation were less likely to deliver at health institution than not-working women or women engaged in any other occupation. This finding is, however, inconsistent with the findings from an earlier study in Ethiopia in which it was observed that occupational status of a mother had no association with institutional delivery service utilization [25]. The observed association seems to suggest that women involved in a job as demanding as agriculture might prefer home delivery to institutional delivery because of limited leisure time. Alternatively, it is possible that women involved in the agricultural occupation are not provided equal opportunity to entertain the right to decision making regarding the place of delivery than their counterparts or they may have lower perceived benefits of institutional delivery.

Among the non-teenage population, multiparous mothers were less likely to visit health facility for delivery. A study carried out in Nepal more than a decade ago [39], in addition to other studies [25,34] showed that multiparity was associated with increased likelihood of home delivery. This pattern can be reasoned by the human behavior of risk-perceiving. Perhaps, a woman who gave a normal vaginal delivery during her earlier deliveries may consider that giving birth is a normal phenomenon and institutional delivery is hence considered unnecessary in her subsequent deliveries.

This study also corroborates with the findings of previous studies regarding the association between ANC visit and its effect on institutional delivery [32,40,41]. Women who made ANC visits had a higher chance of having an institutional delivery. On the first hand, a woman who makes the antenatal visits is generally more health-conscious than a woman with no ANC visit. Such a woman, more often, will continue to follow healthy behaviors and hence deliver in an institution. Additionally, during ANC visits, pregnant women are informed about the risks of home delivery and danger signs of delivery and they are counseled for institutional delivery.

The positive correlation between birth preparedness and institutional delivery among the non-teenage mothers suggest that birth preparedness program prepares pregnant women for institutional delivery. As a part of birth preparedness, pregnant women are more likely to have financial preparations and to be counseled on the danger signs and the health risks associated with home delivery. It could be reasoned that all these planned behaviors work out to address the hindrances (educational and economic) of institutional delivery. This finding is consistent with two earlier studies carried out in two separate locations of Nepal [24,41].

Autonomous women were found to have higher odds of institutional delivery; this finding, however, was only significant among non-teenage mothers. Women autonomy as a strong predictor of institutional delivery was reported from other studies too [42,43]. The linkage between autonomy and institutional delivery service utilization can be explained by the woman’s relative position in the household in terms of decision making. Making a decision regarding the place of delivery is a matter of financial and cultural circumstances [44]. Therefore, if anyone else makes a decision on pregnant woman’s behalf regarding the place of delivery, the health of the mothers might not be a top priority. If the woman herself has a final say regarding delivery place or other health care related matters, she is more likely to make a decision which results in better health outcomes [45]. This might be true in case of choosing the health facility as the place of delivery.

This study has some limitations. Instead of the facility based service utilization data, this study represents the information of service utilization based on the participant’s response. Similarly, other potentially influencing factors for the health service utilization including the quality of health services, and the influence of the community were not included because of the unavailability of such information. Similarly, we have used categorical variable for age, education, etc to make the findings easily interpretable and comparable, which however resulted in the loss of information to some extent.

## Conclusion

This study identified a significant difference in institutional delivery service utilization among the teenage and non-teenage mothers. While the association of most of the background characteristics with institutional delivery was uniform for both teenage and non-teenage mothers, the association with educational status, parity, birth preparedness and women autonomy was significant only for non-teenage mothers. Considering the difference in the interaction of women’s background characteristics with institutional delivery between teenage and non-teenage mothers might help in identifying the pain points and devise targeted interventions to encourage institutional delivery in teenage mothers or non-teenage mothers or both. Future studies may need to focus on exploring why background characteristics behave differently for teenage and non-teenage in determining the institutional delivery service utilization.
